# Alterations to the Gastrointestinal Microbiome Associated with Methamphetamine Use among Young Men who have Sex with Men

**DOI:** 10.1038/s41598-019-51142-8

**Published:** 2019-10-16

**Authors:** Ryan R. Cook, Jennifer A. Fulcher, Nicole H. Tobin, Fan Li, David J. Lee, Cora Woodward, Marjan Javanbakht, Ron Brookmeyer, Steve Shoptaw, Robert Bolan, Grace M. Aldrovandi, Pamina M. Gorbach

**Affiliations:** 1 Department of Epidemiology, Fielding School of Public Health at the University of California, Los Angeles, USA; 2 Divison of Infectious Diseases, Department of Medicine, David Geffen School of Medicine at the University of California, Los Angeles, USA; 3 VA Greater Los Angeles Healthcare System, Los Angeles, USA; 4 Division of Infectious Diseases, Department of Pediatrics, David Geffen School of Medicine at the University of California, Los Angeles, USA; 5 Department of Biostatistics, Fielding School of Public Health at the University of California, Los Angeles, USA; 6 Department of Family Medicine, David Geffen School of Medicine at the University of California, Los Angeles, USA; 7 Department of Psychiatry and Biobehavioral Science, David Geffen School of Medicine at the University of California, Los Angeles, USA; 8 Los Angeles LGBT Center, Los Angeles, USA; 9 Department of Family Medicine, Keck School of Medicine at the University of Southern California, Los Angeles, USA

**Keywords:** Inflammation, Epidemiology, Preclinical research

## Abstract

Methamphetamine (MA) use is a major public health problem in the United States, especially among people living with HIV (PLWH). Many MA-induced neurotoxic effects are mediated by inflammation and gut microbiota may play a role in this process, yet the effects of MA on the microbiome have not been adequately explored. Therefore, we performed 16S rRNA gene sequencing on rectal swab samples from 381 men who have sex with men, 48% of whom were PLWH and 41% of whom used MA. We compared microbiome composition between MA users and non-users while testing for potential interactions with HIV and controlling for numerous confounders using inverse probability of treatment weighting. We found that MA use explained significant variation in overall composition (R^2^ = 0.005, *p* = 0.008) and was associated with elevated *Finegoldia, Parvimonas, Peptoniphilus*, and *Porphyromonas* and reduced *Butyricicoccus* and *Faecalibacterium*, among others. Genera including *Actinomyces* and *Streptobacillus* interacted with HIV status, such that they were increased in HIV+ MA users. *Finegoldia* and *Peptoniphilus* increased with increasing frequency of MA use, among others. In summary, MA use was associated with a microbial imbalance favoring pro-inflammatory bacteria, including some with neuroactive potential and others that have previously been associated with poor HIV outcomes.

## Introduction

The 2017 National Survey on Drug Use and Health estimated that nearly one million people in the United States were current users of methamphetamine (MA) or had a MA use disorder in the past year^1^. MA use is a major public health concern with myriad negative health consequences ranging from anxiety and confusion to psychosis and violent behavior; chronic abuse may even result in severe and lasting structural changes in the brain affecting emotional regulation and cognition^2^. MA use is much more prevalent among people living with HIV, with rates of recent use 30 times higher and rates of dependence 33 times higher than the general population (0.3% vs. 9% recent use and 0.4% vs. 13% dependence)^1,3,4^. MA increases susceptibility to HIV infection by altering immune activity^5,6^ inhibiting neurocognitive processes involved in judgement and decision-making^7^ and increasing the frequency of risky sex acts^4,8^. MA use among people living with HIV is associated with reduced likelihood of achieving viral suppression^9^, faster disease progression^10^, and increased risk of transmission to others^11^.

Many MA-induced neurotoxic effects are mediated by inflammation^5,12^, and the microbiome, which is involved in inducing and regulating the immune system^13^, may play a role in this process. Exposure to MA impacts both innate and adaptive immunity, increasing production of inflammatory cytokines, inhibiting T-cell proliferation, altering gene expression of immune cells, modifying cytokine signaling pathways, and increasing blood-brain barrier permeability^5,14,15^. MA damages gut wall integrity and increases intestinal permeability leading to the translocation of microbiota into the body^15^. This process disrupts symbiotic interactions between the host immune system and microbiota, inducing an immune response that may cyclically exacerbate intestinal permeability and further inflammation^16^. Microbial translocation has been cited as one of the key drivers of chronic inflammation described in many other diseases, including HIV, and may play a similar role in MA-induced inflammation and toxicity. Furthermore, mediated by inflammation, disruption of gut bacterial communities (termed “dysbiosis”) may be a mechanistic link between methamphetamine use and HIV transmission and disease progression.

Dysbiosis has been described in individuals with substance use disorders^17^, chronic prescription opioid^18^ and cocaine users^19^, as well as people living with HIV^20^ and those practicing anal intercourse^21^. We recently showed that MA use was associated with microbiome changes in a small sample of HIV-infected MSM^22^. However, no large studies into the effects of MA on the microbiome have been completed, and no studies have examined the potential role of HIV in MA-induced dysbiosis. In order to address this gap, we studied the effects of MA on the gastrointestinal microbiome in a cohort of young men who have sex with men (MSM), approximately half of whom were HIV-infected, and all of whom were engaging in anal intercourse. We hypothesized that MA use would be associated with increased relative abundance of pro-inflammatory and pathogenic bacterial taxa as well as alterations to those with neurologic effects. We also hypothesized that MA use and HIV would interact to increase the severity of dysbiosis.

## Materials and Methods

### Study population

Specimens and data for this study were drawn from a larger cohort, the NIDA-funded Minority Men who have Sex with Men Cohort at UCLA Linking Infections Noting Effects (MASCULINE, or mSTUDY). Subject selection procedures have been previously described^23^. Briefly, participants were all men born males, aged 18–45, with approximately one-half of the sample purposefully included due to current substance use (the other half non-substance users) and one-half of the sample purposefully included due to HIV-infection (the other half being HIV-negative). The mSTUDY was approved by the UCLA South General Institutional Review Board (IRB), and the current study was approved by the UCLA Medical IRB 1. All participants provided written informed consent prior to participation and all study procedures were done in accordance with ethical principles for human subjects research.

### Specimen collection and DNA preparation

As previously described^23^, samples included in this study were rectal swabs (FLOQSwabs, Copan Diagnostics, Murrieta, CA). The majority (76%) were collected via anoscopy under direct mucosal visualization and without preparatory enema at approximately 8 cm from the anal verge. Due to a protocol change, others (24%) were participant self-collected at approximately 4–5 cm from the anal verge. Collection method was taken into account in the analysis (Table 1; Supplemental content). Swabs were immediately frozen neat at −80 °C until processing in bulk. For DNA processing the samples were transferred to Lysing Matrix E tubes (MP Biomedicals, Burlingame, CA) containing RLT lysis buffer (Qiagen, Hilden, Germany) and bead-beated on a TissueLyser (Qiagen). DNA was then extracted using the AllPrep DNA/RNA/Protein kit (Qiagen) per manufacturer’s protocol.

**Table 1:** Participant characteristics, N = 381 men who have sex with men in Los Angeles, CA.

	MA- negative n = 225 mean (sd), median n (%)	MA-positive n = 156	*P* ^d^	SMD^e^ (pre, post IPTW)
Age	30.17 (6.85), 29	32.58 (6.75), 33	<0.001	0.35, 0.16
HIV+	80 (35.6)	102 (65.4)	<0.001	N/A
Race/ethnicity			0.6	0.1, 0.07
Black-Non Hispanic	93 (41.3)	57 (36.5)		
Hispanic	107 (47.6)	79 (50.6)		
Other-Non Hispanic	25 (11.1)	20 (12.8)		
Homeless in past 6 months	52 (23.1)	77 (49.4)	<0.001	0.57, 0.28
Had RAI in last 7 days	102 (45.3)	65 (41.7)	0.5	0.07, 0.03
Number of RAI acts in past month	2.09 (4.94), 0	2.88 (5.33), 1	<0.001	0.15, 0.03
Number of anal sex partners in past 6 months	6.17 (7.60), 3	8.79 (9.28), 5	<0.001	0.31, 0.18
Positive for STI^a^	19 (8.4)	28 (17.9)	0.006	0.28, 0.18
Marijuana use in past 6 months			0.1	0.23, 0.21
Daily/Weekly	69 (30.7)	60 (38.5)		
Monthly/less	52 (23.1)	41 (26.3)		
Never	104 (46.2)	55 (35.3)		
Cocaine use in past 6 months	40 (17.8)	60 (38.5)	<0.001	0.47, 0.24
Tobacco smoker	73 (32.4)	95 (60.9)	<0.001	0.6, 0.38
Binge drinking in past 6 months^b^	138 (61.3)	91 (58.3)	0.6	0.06, 0.04
Antibiotic use in past month	15 (6.7)	16 (10.3)	0.2	0.13, 0.07
Sample collection strategy			0.5	0.07, 0.01
Anoscopy	169 (75.1)	122 (78.2)		
Self-collected	56 (24.9)	34 (21.8)		
Type of ART			<0.001	0.56, 0.28
INSTI + NRTI	30 (13.3)	39 (25.0)		
NNRTI + NRTI	25 (11.1)	23 (14.7)		
NRTI + PI	15 (6.7)	15 (9.6)		
Other	4 (1.8)	12 (7.7)		
HIV+ and missing ART data	6 (2.7)	14 (9.0)		
HIV− pre-exposure prophylaxis (PrEP) user	30 (13.3)	7 (4.5)		
HIV−, no PrEP	115 (51.1)	47 (30.1)		
Among HIV+ participants only				
HIV RNA log_10_ copies/mL (median, IQR) c	1.03 (0.7)	1.03 (1.7)		N/A
CD4 cells/mm^3^ (median, IQR)^c^	590.5 (267)	635 (424.3)		N/A
CD4 cells/mm^3^ <200	5 (2.2)	9 (5.8)		0.18, 0.15

MA = Methamphetamine; SMD = Standardized mean difference; RAI = Receptive anal intercourse; STI = Sexually transmitted infection; ART = Antiretroviral therapy; INSTI = Integrase strand transfer inhibitor; NRTI = Nucleoside reverse transcriptase inhibitor; NNRTI = Non-nucleoside reverse transcriptase inhibitor; PI = Protease inhibitor.

^a^Sexually transmitted infections include rectal gonorrhea, rectal chlamydia as well as primary/secondary syphilis.

^b^Binge drinking defined as 6 or more drinks on one occasion.

^c^HIV status, HIV RNA, and continuous CD4 cell count were not included in the inverse probability of treatment weight model, all other variables in the table were included. HIV status was taken into account in the analyses by stratifying on it (if there was evidence for an interaction between MA and HIV) or conditioning on it (if there was no evidence for an interaction).

^d^*p* values are from Wilcoxon tests or Chi-square tests.

^e^SMD is a measure of imbalance across groups; higher SMDs indicate greater imbalance. Average SMD before weighting = 0.28, after weighting = 0.14.

### 16S rRNA gene sequencing and data processing

Microbiome profiling was performed by sequencing of the V4 region of the 16S rRNA gene as previously described^23–25^. Briefly, the V4 region was amplified in triplicate reactions using Golay-barcoded primers 515F/806R. PCR products were then pooled and sequenced on the Illumina MiSeq platform using 2 × 150 bp v2 chemistry. The sequences were demultiplexed with Golay error correction using QIIME v1.9.1^26^, and Divisive Amplicon Denoising Algorithm (DADA2) version 1.8 was used for error correction, exact sequence inference, read merging, and chimera removal^27^. The resultant amplicon sequence variant (ASV) table comprised 19,955,039 total merged read pairs (mean per sample = 52,375; range 10,906 to 124,889). Taxonomic assignment was performed using RDP trainset 16 (10.5281/zenodo.810827). Rarefaction was performed at a depth of 10,906 reads for alpha diversity analyses. To normalize all other analyses, estimates of relative library sizes (“size factors”) were obtained by calculating geometric means of pairwise read count ratios^28^.

### Measurement of MA use

MA use was measured using an adapted version of the NIDA-modified ASSIST^29^. Participants were asked how often they used MA in the previous six months; response choices were “Daily”, “Weekly”, “Monthly”, “Less often”, “Once”, and “Never.” For most analyses we categorized participants as MA users if they indicated any use in the past six months, and non-users if they responded “Never.” For the dose-response analysis, we combined “Monthly”, “Less often”, and “Once” into “Monthly or less often,” given that infrequent exposures are likely to have similar effects on the microbiome. In addition, participants were screened for MA use via urinalysis [Fastect® II (Branan Medical Corporation); iScreen® Dip Card (Alere)]. We did not use the urinalysis results as our primary exposure variable because the detection window for MA is 48–72 hours and no exposure quantification (and thus dose-response analysis) could be done. We instead compared the self-report and urinalysis results as a sensitivity analysis (Supplementary Figs S4, S5 and S6). HIV status was ascertained upon entry into the cohort by medical record review (for known HIV-infected participants) or the OraQuick Advance® HIV 1/2 (OraSure Technologies, Bethlehem, PA).

### Behavioral and clinical covariates

Analyses controlled for a large set of behavioral and clinical covariates including age, race/ethnicity, homelessness in past six months, number of receptive anal intercourse (RAI) acts in past month, number of anal sex partners in past six months, an indicator for RAI in the past seven days, an indicator for a positive STI test (including PCR tests for rectal gonorrhea and chlamydia and serology for primary or secondary syphilis), self-reported use of marijuana and cocaine, tobacco smoking, and binge drinking (defined as 6+ drinks on more than one occasion) in the past six months, and use of antibiotics in the past month. We also controlled for type of antiretroviral therapy (including use of pre-exposure prophylaxis if HIV−) and an indicator for CD4 cell count <200 (Table 1). Measures and assays have been previously described^23^.

### Statistical analyses

We compared clinical and behavioral characteristics between MA users and non-users using descriptive statistics, Wilcoxon or Chi-square tests, and standardized mean differences (see Table 1; Supplemental content). All analyses of microbiome outcomes were adjusted for clinical and behavioral confounders using inverse probability of treatment weighting (IPTW). IPTW is a technique in which the study sample is re-weighted to achieve balance between exposure groups (here, MA users vs. non-users) on important covariates so that their confounding effects are substantially reduced^30^. Covariates included in the IPTW model are listed in Table 1, and further information about the IPTW calculation and adjustment process is available in the supplemental content as well as our previous publication^23^. Weights were estimated using generalized boosted models (R package ‘twang’^31^) and robust standard errors for IPTW-adjusted analyses were obtained using the sandwich estimator (R package ‘sandwich’^32^). All analyses in this study proceeded by first testing for interactions between MA use and HIV status using multiplicative interaction terms. An alpha level of 0.1 was used as a cutoff for significance of interaction tests; if significant, comparisons of MA users vs. non-users are presented stratified by HIV status (retaining HIV status and the interaction term in the model). If no significant interaction was detected, comparisons of MA users vs. non-users were completed controlling for HIV status (retaining HIV status as a covariate but dropping the interaction term).

The R package ‘phyloseq’^33^ was used to calculate distance matrices, alpha diversity metrics, and for ordination. Permutational multivariate ANOVA (PERMANOVA; R package ‘vegan’^34^) was used to test for differences in overall microbial composition. Cluster analysis was performed using the partitioning around mediods method with the optimal number of clusters chosen according to the Calinski-Harabasz statistic (R packages ‘cluster’^35^ and ‘fpc’^36^). Logistic regression was used to assess the relationship between MA use and cluster membership. Linear regression was used to test for differences in alpha diversity between MA users and non-users. Zero-inflated negative binomial (ZINB) models were used to test for differences in individual bacterial genera between groups (R package ‘pscl’^37^). We employed a previously described model selection strategy^23^ to choose the optimal ZINB model for each genus. A pre-filtering step excluded genera appearing in less than 10% of samples as well as those with less than 100 total reads across all samples, resulting in 78 genera included in ZINB analyses. Dose-response analysis was completed by regressing bacterial counts on frequency of MA use using orthogonal polynomial coding of linear and quadratic curves. As sensitivity analyses, all analyses were repeated redefining MA use according to urine drug screen results (except for dose-response, owing to the qualitative nature of urine drug screening). Finally, we examined short-term interactions between MA use and the microbiome using a correlation analysis among individuals testing positive for MA on urine drug screen. Correlations were calculated with the sparCC method^38^ with bootstrapped standard errors (R package ‘SpiecEasi’^39^).

PERMANOVA, alpha diversity, and dose-response analyses utilized a threshold of *p* < 0.05 to determine statistical significance. In order to account for the large amount of genera tested, *p* values obtained in ZINB analyses and sparCC correlations were corrected using Benjamini & Hochberg’s False Discovery Rate (FDR) method^40^. FDR-adjusted p values are labelled as *q* values, and *q* < 0.1 was used as a threshold to determine statistical significance. Accordingly, we display 90% false coverage rate (FCR)-adjusted confidence intervals^41^ to accompany these analyses. All statistical analyses were performed using R v.3.5.1^42^ and graphics were generated with ggplot2^43^.

## Results

### Participant characteristics

This study included 381 participants, 156 MA users (41%) and 225 non-users (59%). All participants were MSM, the mean age was 31, and most were Hispanic (49%) or non-Hispanic Black (39%). One hundred eighty-two participants were HIV+ (48%); sixty-five percent of MA users were HIV+ as compared to 36% of non-users. MA users were also older than non-users, were more likely to have experienced homelessness, had RAI more frequently, had more anal sex partners, were more likely to have recently used cocaine, and were more likely to be tobacco smokers (Table 1).

### Effects of MA use on overall microbial composition and diversity

**Figure 1:**
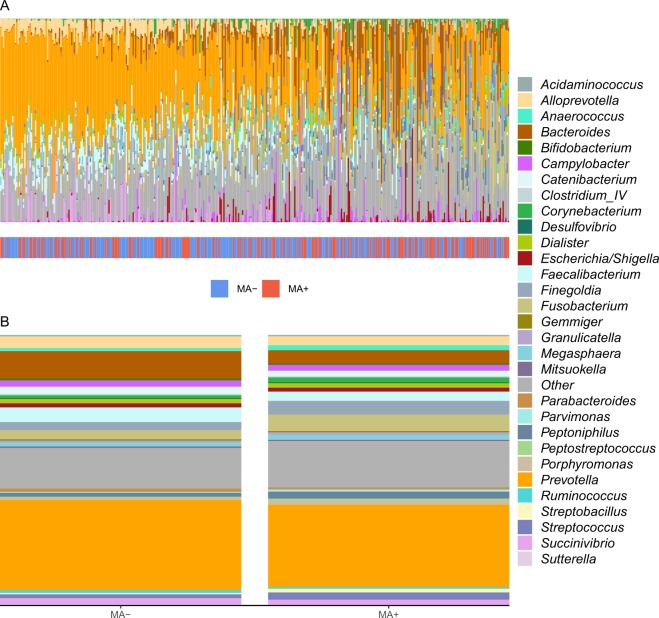
Rectal microbial composition of study participants, N = 381. (**A**) Columns represent the relative composition of each subject’s microbiome at the genus level. Methamphetamine (MA) use by the subjects is indicated by a colored line below their composition. Subjects are ordered by the first principal coordinate of a Bray-Curtis pairwise distance matrix. Genera representing less than 1% of the composition on average across samples were combined into “Other.” (**B**) Average microbial composition within each MA use group. Bacterial genera representing less than 1% of the overall relative composition or present in less than 10% of the samples were grouped into “Other.”

**Figure 2:**
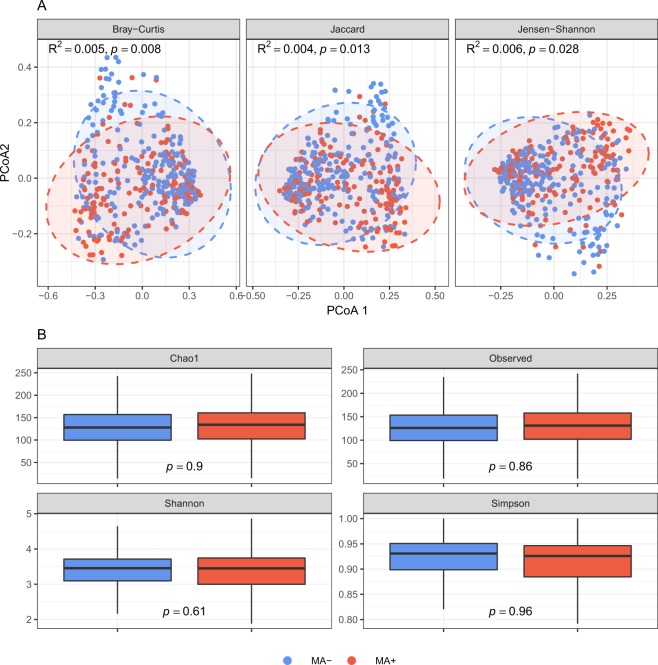
Associations between methamphetamine (MA) use and overall microbial composition and diversity. (**A**) Ordination of the samples using principal coordinates analysis. PCoA = Principal coordinate axis. Ellipses are 95% confidence regions for each group assuming points follow a multivariate t distribution. R^2^ and *p* values are from PERMANOVA analyses of distance metrics. (**B**) Boxplots of diversity metrics. Boxes represent the inverse probability of treatment weight-adjusted lower, median, and upper quartiles of the data and whiskers are 1.5*interquartile range. *p* values are from IPTW-adjusted linear regression analyses comparing diversity metrics between MA users and non-users.

PERMANOVA analyses with Bray-Curtis, Jaccard, and Jensen-Shannon distances did not reveal significant evidence supporting an interaction between MA and HIV on overall microbial composition (all *p* > 0.1; interaction *p* values supplied in Supplementary Table S1). Therefore, we described and compared overall composition between MA users and non-users while controlling for HIV status. Qualitative examination of descriptive barplots suggested increased *Finegoldia, Fusobacterium, Peptoniphilus, Porphyromonas, Streptobacillus*, and *Streptococcus* and decreased *Bacteroides, Faecalibacterium*, and *Succinivibrio* in MA users compared to non-users (Fig. 1A,B). Ordination of Bray-Curtis, Jaccard, and Jensen-Shannon distances by principal coordinates analyses revealed clustering by MA status (Fig. 2A). These findings were supported by PERMANOVA analyses showing that MA explained significant variation in overall microbial composition (Bray-Curtis R^2^ = 0.005, *p* = 0.008; additional results in Fig. 2A). Additionally, a clustering analysis using partitioning around medoids of Bray-Curtis distances revealed two distinct clusters in the microbiome data, which were significantly related to MA use. Adjusted for HIV status and our behavioral and clinical covariate set, the likelihood of classification into the second cluster was 2.24 times higher for MA users compared to non-users (OR = 2.24, *p =* 0.011, pseudo-R^2^ = 0.008).

No significant interactions between MA and HIV were detected in observed diversity or Chao1, Shannon, or Simpson indices (all *p* > 0.1; Supplementary Table S1), and no differences in diversity were detected between MA users and non-users in any metric (Fig. 2B). Despite lack of evidence for an interaction between HIV and MA use, we display descriptive, ordination and alpha diversity plots stratified by HIV status in Supplementary Figs S1 and S2.

### Effects of MA use on specific genera

**Figure 3:**
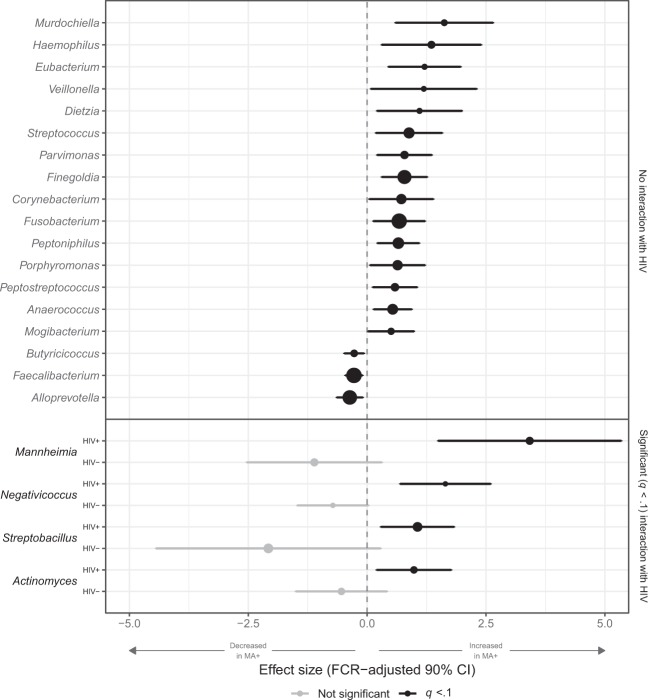
Comparisons of individual genera between methamphetamine (MA) users and non-users. Forest plots of results of zero-inflated negative binomial models comparing genus-level bacterial counts between methamphetamine (MA) users and non-users. Inverse probability of treatment-weighted effect sizes (log normalized count ratios) and false coverage rate (FCR)-adjusted 90% confidence intervals are plotted, with statistical significance (*q* < 0.1) indicated in black. Genera with no effect are not shown. Dots are sized proportionally to overall mean abundance across samples, i.e., genera with larger dots are, on average, more abundant.

Using ZINB models with IPTW adjustment, we found differences between MA users and non-users in multiple genera. For some, there was no evidence for an interaction between MA and HIV: Regardless of HIV status, MA users had higher levels of *Finegoldia, Fusobacterium, Parvimonas, Peptoniphilus, Peptostreptococcus*, and *Porphyromonas*, and lower levels of *Butyricicoccus* and *Faecalibacterium*, among others (all *q* < 0.1; Fig. 3; estimates and *q* values supplied in Supplementary Table S2). For four genera, a significant (*q* < 0.1; interaction *q* values supplied in Supplementary Table S2) interaction between HIV and MA was detected. *Actinomyces, Mannheimia, Negativicoccus*, and *Streptobacillus* were increased in HIV+ MA users compared to HIV+ non-users, but no difference was found in the absence of HIV. No genera were significant only in the HIV− stratum.

### Dose-response analysis of bacterial counts on increasing frequency of MA use

Of the 156 MA users in the study, 40 were daily users, 35 used weekly, and 81 used monthly or less often. Counts of *Anaerococcus, Corynebacterium, Dietzia, Finegoldia, Mannheimia* and *Peptoniphilus* increased linearly with increasing frequency of MA use (*q* < 0.1 for test of linear trend). No significant quadratic dose-response curves were noted in any genera (Fig. S3).

### Sensitivity analysis using urine toxicology screening to define MA use

Our findings, which were based on participant self-report of MA use, were consistent when we re-defined MA use using urine drug screening results. All participants completed urine drug screening, and fourteen percent of study participants (n = 52) tested positive for MA including 3 individuals who self-reported no MA use (49/52 who tested positive also self-reported using MA). One hundred seven self-reported using MA, but tested negative, likely because their last use was outside the drug screen window of detection. HIV+ participants were more likely to have a positive MA urine drug screen (21% among HIV+ vs. 7% among HIV−, p < 0.001).

In biomarker analyses, MA use was still a significant driver of variation in overall microbial composition (Bray-Curtis R^2^ = 0.008, *p* = 0.008), and no differences in alpha diversity were noted between MA users and non-users. Many of the same genera were elevated in MA users, e.g., *Finegoldia, Fusobacterium, Peptoniphilus, Peptostreptococcus*, and *Porphyromonas*, and elevations in *Streptobacillus* in the HIV+ stratum were noted in both analyses. Depletion in *Faecalibacterium* was consistent across analyses; the biomarker analysis also identified depletions in *Clostridium* cluster XI and *Lactobacillus* in MA users. Results from this sensitivity analysis are presented in Supplementary Figs S4–S6.

Finally, to examine short-term interactions between MA use and the microbiome, we performed a genera correlation analysis among individuals testing positive for recent MA use. Large, significant positive correlations were noted between *Parvimonas* and *Campylobacter* as well as *Peptoniphilus* and *Campylobacter*, among others (sparCC correlation >0.6 and *q* < 0.1). Moderate-to-large negative correlations were noted between *Alloprevotella* and *Butyricicoccus* as well as *Parabacteroides* and *Campylobacter*, among others (sparCC correlation < −0.4 and *q* < 0.1). Full results are presented in Supplemental Table S3 and Fig. S7.

## Discussion

This study of 381 MSM who were either HIV-infected or at high risk for HIV acquisition found that MA use significantly impacted gut microbial composition after controlling for multiple clinical and behavioral confounders. Measures of overall composition were altered by MA use, but measures of diversity were not, and the associations between MA and overall composition and diversity did not depend on participants’ HIV status. Several genera were increased in MA users regardless of HIV status, many of them considered pro-inflammatory and pathogenic, while others were increased among HIV+ participants only. We found that the abundance of some pro-inflammatory taxa increased and commensals decreased with increasing frequency of MA use. Finally, we were able to replicate our findings using a biomarker confirming recent MA use (urine drug screen). MA effect sizes were slightly larger in the biomarker analysis, likely because the window of detection for MA is short, making frequent users more likely to test positive. Our analyses utilized a novel method of confounder control, IPTW, to account for several factors that have previously been associated with dysbiosis (e.g. RAI^21^, cocaine use^19^, and alcohol use^44^), making our findings more likely to be truly attributable to MA use.

Although little research has been done on the effects of MA on the microbiome, our results are mostly consistent with previously published literature. A study of 37 HIV+ individuals from the same cohort as the current data^22^ (none of the individuals in this current sample were included in the previous study) reported a MA effect size (PERMANOVA R^2^) of 0.1, larger than the effect we found. As in our study, there were no significant differences in diversity between MA users and non-users. Enrichment in *Porphyromonas* in MA users was consistent across studies. As a well-known modifier of inflammatory cytokines and a potential cause of intestinal permeability^45^, *Porphyromonas* may play a role in MA-associated inflammation and deserves further investigation. Another study in which MA was administered to rats reported an overall effect of MA that is consistent with our findings (R^2^ of 0.008)^46^. This study also reported higher alpha diversity in the MA-conditioned group, which was not replicated in our study, and taxonomic differences that do not overlap with our findings, likely because of differences between the mouse model and a human cohort. Finally, a study comparing individuals with substance use disorders (SUDs) to healthy controls found a large effect of SUD (R^2^ of 0.067), higher observed diversity among individuals with SUDs, and differences in specific genera that do not match our findings^17^. However, participants with MA use disorder only accounted for 30% of the SUD group, and the study did not control for large differences in lifestyle and clinical confounders between individuals with SUDs and healthy controls.

MA use is associated with increases in production and alterations in gene expression of many pro-inflammatory cytokines^6,47^, which may contribute to neurological deficits, anxiety, and impaired memory^48^. Many of the bacterial genera that were elevated in MA users, such as *Porphyromonas*^45^, *Veillonella*^49^, and *Fusobacterium*^50^ have been correlated with increases in pro-inflammatory cytokines. MA also exacerbates systemic inflammation by damaging gastrointestinal barrier integrity and inducing permeability, allowing the translocation of microbes and microbial products into the body. Our study identified depletions in the butyrate-producing genera *Faecalibacterium* and *Butyricicoccus* in MA users, which have been inversely correlated with biomarkers of microbial translocation^51,52^. A study of patients with alcohol use disorder showed that those with higher levels of gut permeability had lower levels of *Bifidobacterium* and *Faecalibacterium* species and exhibited more symptoms of alcohol dependence and cravings^53^.

Emerging preclinical research has demonstrated a complex interplay between the microbiome and the central nervous system^54^, leading to inquiries about the role of dysbiosis in addiction pathology. Gut bacteria produce neuroactive substances, including serotonin, epinephrine and dopamine^55^, which may access the brain’s reward centers via gut-innervating vagal neurons^56^. *Streptococcus*, which was elevated in MA users in our study, can produce serotonin and *Lactobacillus*, which was depleted in MA users (in our biomarker analysis), can produce gamma-aminobutyric acid (GABA)^55^. Laboratory experiments of other drugs of abuse have suggested a connection between dysbiosis and addiction pathology. For example, increased sensitivity to cocaine reward through alterations in dopaminergic pathways has been observed in mice with experimentally disrupted microbiome^57^. Another study showed that manipulation of the microbiome resulted in several characteristics of opioid dependence in mice, such as reduced opioid analgesic potency and impaired reward behavior^58^. In addition, there is some preclinical evidence that “repairing” the microbiome might alleviate addiction pathology, e.g., *Lactobacillus* species restored chemically-depressed dopamine levels in the prefrontal cortex when administered to rats as a probiotic^59^. However, these studies have not involved MA, and the preclinical connection between dysbosis and MA dependence is speculative.

In humans, potential mechanisms linking dysbiosis to the pathology of MA dependence remain largely unexplored. There is limited clinical evidence linking dysbiosis with symptoms associated with MA dependence, such altered stress response and increased depression. Butyrate-producing *Faecalibacterium*, which was decreased in MA users in our study, has been associated with reduced depression and higher quality of life^60^. Common symptoms of MA withdrawal, including depression, anxiety, and fatigue, have been correlated with imbalances in gut microbiota^61^, and probiotics have been used to successfully reduce anxiety and depression in clinical trials^62^. It is plausible that targeting dysbiosis may ease these symptoms among individuals undergoing treatment for MA use disorders.

We also found a number of genera that were impacted by MA which have previously been shown to play a role in HIV acquisition, disease progression, and pathogenesis. Increased abundances of *Finegoldia* and *Peptoniphilus* in the penile microbiome have been associated with elevated risk for HIV seroconversion in men^63^, and *Parvimonas* has been shown to increase genital tract inflammation^64^ and the risk of HIV acquisition in women^65^. The implications of enrichment of these bacteria in the rectal microbiome have not been explored; however, it is likely that inflammation exacerbated by dysbiosis underlies the increase in seroconversion risk, which would be highly relevant to at-risk MSM. We also found that MA use increased *Fusobacterium*, which has been correlated with decreased CD4+ T-cell count and increased T-cell activation in HIV+ individuals as well as reduced T-cell recovery following ART initiation^66^. HIV and MA interacted to multiplicatively increase *Actinomyces*, which may play a role in reactivating HIV in latently infected cells^67^. *Parvimonas* and *Peptostreptococcus* are oral pathogens that have been implicated in periodontal infections among HIV+ individuals^68,69^, and their damaging effects may be heightened in MA users due to MA’s proclivity to reduce saliva production. Finally, increased abundance of *Veillonella* has been linked with HIV-associated pulmonary diseases^70^. Our study, showing that MA impacted the relative abundance of each of these genera, may highlight mechanisms underlying the relationship between MA use, HIV acquisition and transmission risk, and HIV disease progression which warrant further investigation.

Our results should be interpreted considering the following limitations. Primarily, no diet data is collected for this cohort. We controlled for race/ethnicity and homelessness, which may impact diet and thus mitigate the effects of this limitation; however, we were unable to fully account for the effects of diet in our analyses. Using IPTW, our study accounts for a plethora of other clinical and behavioral confounders which may have masked true findings or generated spurious associations in previous studies. However, IPTW cannot achieve perfect balance between exposure groups in real-world research applications, and thus we cannot rule out residual confounding even by variables included in our analyses. Our study was also conducted in a cohort comprised entirely of MSM, all of whom were practicing anal intercourse, which increases internal validity by eliminating the effects of some important confounders (e.g. gender, sexual behavior). However, this may limit the generalizability of our findings to other groups, such as women who use MA. Finally, because the ability of 16 S gene sequencing to identify bacterial species is limited, we conducted our analyses at the genus level. We caution that differences in genera do not necessarily correspond to differences in functionally important species.

MA use remains a significant public health challenge, especially among people living with HIV. Our study found that MA use was associated with an imbalance in gut microbial composition favoring pro-inflammatory, potentially pathogenic bacteria, including some with neuroactive potential. There is currently no accepted pharmaceutical treatment for MA use disorder and limited evidence for the effectiveness of cognitive-behavioral therapy; further research into changes in the microbiome associated with MA use may inform therapeutic approaches for individuals with MA use disorder. Moreover, increases in multiple taxa that have been previously associated with poor HIV outcomes or HIV transmission and acquisition are particularly concerning in our study population of MSM who were either HIV-infected or at high risk for infection. Additional investigation into the mechanisms underlying these associations may improve HIV prognosis and prevent future infections among this vulnerable group.

## Supplementary information

Supplemental content

## Data Availability

The datasets generated during and/or analysed during the current study are available from the corresponding author on reasonable request. All sequencing data has been deposited into BioProject with the accession number PRJNA422134.
