# Supplementary material for: Alterations to the Gastrointestinal Microbiome Associated with Methamphetamine Use among Young Men who have Sex with Men
Source: Sci Rep. 2019 Oct 16;9:14840. doi: 10.1038/s41598-019-51142-8 (PMC6795845; doi:10.1038/s41598-019-51142-8)
Supplement: Supplementary file 1 — Supplemental content [file 41598_2019_51142_MOESM1_ESM.docx]

Alterations to the Gastrointestinal Microbiome Associated with Methamphetamine Use among Young Men who have Sex with Men

Ryan R. Cook, Jennifer A. Fulcher, Nicole H. Tobin, Fan Li, David J. Lee, Cora Woodward, Marjan Javanbakht, Ron Brookmeyer, Steve Shoptaw, Robert Bolan, Grace M. Aldrovandi, Pamina M. Gorbach

***Inverse probability of treatment weight (IPTW) calculation***

Inverse probability of treatment weighting (IPTW) is a balancing procedure used to render the treatment or exposure under study (here, MA use) independent of confounding variables. In essence, weighting by the inverse probability of treatment/exposure transforms the study sample into a “pseudo-population” where exposure status is independent of variables used in the IPTW calculation. It has been shown that if the model used to estimate the weights includes all relevant confounders and is not misspecified, in a weighted population, the effect estimate of exposure on outcome is unbiased.^1^ With IPTW, the outcome models are “marginal structural models” instead of conditional on covariates, as in multiple regression. Modeling microbiome data marginally offers several advantages including the ability to control for many confounding factors without inducing overfitting bias^2^ or losing efficiency due to overstratification.^3^

For a two-level exposure variable *X*, the IPTW is defined as $\frac{P(X=x)}{P(X=x|\boldsymbol{C})}$ where *x* equals 0 or 1, unexposed or exposed, and ***C*** is a (possibly high-dimensional) set of confounding variables. In this paper, we consider potential interactions between two such variables: HIV status and MA use. As outlined by VanderWeele (2009),^4^ the IPTW needed for estimation of the effects of two exposures depends on whether one wishes to study effect modification (the effect of one exposure within strata of another) or interaction (the joint, simultaneous, or combined effects of both exposures). In this study we examine the effects of MA use on the microbiome within strata of HIV status (effect modification); accordingly, IPTW were defined as $\frac{P(M=m|H=h)}{P(M=m|H=h, \boldsymbol{C})}$ for MA use status *M* (user, non-user)*,* HIV status *H* (HIV+, HIV-)*,* and set of covariates ***C***. For a list of covariates included in ***C***, see Table 1. For the dose-response analyses, where MA use was treated as a four-category variable, IPTW were defined as $\frac{P(M=m)}{P(M=m|\boldsymbol{C})}$ for MA use frequency *M* $\in$ {Never, Monthly or less, Weekly, Daily}. HIV status was included as an additional covariate in dose-response ZINB models. IPTW were estimated using generalized boosted models.^5^

In theory, weighting by the inverse probability of treatment will perfectly balance all covariates used to create the weights across exposure groups, thereby eliminating the possibility that these covariates can act as confounders. Because of finite sample sizes and the need for parametric models, perfect balance is never achieved in practice, except in the most trivial cases. The absolute value of the standardized mean difference (SMD), which is the mean difference in a covariate between groups in units of its standard deviation, is a metric commonly used to measure the performance of IPTW estimates. SMDs for each covariate in the IPTW model are calculated before and after weighting, and reductions in covariate-specific SMDs represent reductions in confounding by that covariate. A substantial reduction in the average SMD across all covariates indicates the IPTW, when applied during the exposure-outcome analysis, will significantly reduce confounding. There is no agreed-upon “cut-off” SMD value indicating adequate confounding control; some have suggested 0.1,^6^ but others have indicated that is probably unnecessarily conservative.^7^ Table 1 shows the SMD for each covariate included in the IPTW model, before and after weighting, as well as the average SMD before and after applying the weights. Prior to weighting, the average SMD for balance across MA use was 0.28; after applying IPTW, it was 0.14.

***Impact of collection strategy on microbiome characterization***

Sample collection, storage, and processing strategies may have a significant impact on microbiome characterization.^8^ In order to streamline visits, control costs, and reduce participant burden, the mSTUDY protocol for collecting rectal swab samples changed from anoscopy to self-collection in December 2016. Approximately one-quarter of samples included in this study were participant self-collected. Using Bray-Curtis distances, prior to adjustment for covariates, collection method explains 0.43% of the variability in overall microbiome composition (R2 = .0043, p = .034). With adjustment for study covariates, collection method explains 0.39% of the variability in the microbiome (R2 = .0039, p = .051). However, even though collection method has a significant impact on microbiome characterization, it is highly unlikely to confound our results, as rates of each collection strategy were very similar between exposure groups. Prior to IPTW adjustment, the differences between study groups were non-significant (3% difference between groups, p = .49); following adjustment, differences are even further reduced (0.1% difference between groups, p = .98). See Table 1 for additional details.

Table S1. *p* values for interaction between HIV and MA use

| Overall composition (PERMANOVA) | |
| --- | --- |
| Statistic | Interaction *p* value |
| Bray-Curtis | .193 |
| Jaccard | .252 |
| Jensen-Shannon | .115 |
| Alpha diversity | |
| Statistic | Interaction *p* value |
| Observed | .236 |
| Chao1 | .285 |
| Shannon | .465 |
| Simpson | .909 |

Table S2. Results of differential abundance tests of bacterial genera between MA users and non-users. Tests for multiplicative interaction between MA use and HIV status were performed; if significant (*q* < .1), results are presented stratified by HIV status. Estimates are log normalized ratios of bacterial counts adjusted for confounding using inverse-probability of treatment weighting (IPTW).

| Taxonomy | Interaction *q* value | Stratum | Estimate | SE | *p* | *q* |
| --- | --- | --- | --- | --- | --- | --- |
| Acetanaerobacterium | 0.732 |  | 0.50 | 0.27 | 0.067 | 0.198 |
| Acidaminococcus | 0.873 |  | -0.11 | 0.10 | 0.268 | 0.532 |
| Actinomyces | 0.095 | HIV- | -0.54 | 0.43 | 0.212 | 0.463 |
|  |  | HIV+ | 0.98 | 0.35 | 0.005 | 0.030 |
| Alistipes | 0.801 |  | 0.04 | 0.31 | 0.903 | 0.940 |
| Allisonella | 0.924 |  | 0.02 | 0.13 | 0.906 | 0.940 |
| Alloprevotella | 0.791 |  | -0.36 | 0.12 | 0.004 | 0.026 |
| Anaerococcus | 0.873 |  | 0.54 | 0.18 | 0.003 | 0.026 |
| Arcanobacterium | 0.924 |  | 0.75 | 0.46 | 0.105 | 0.281 |
| Bacteroides | 0.786 |  | -0.18 | 0.20 | 0.353 | 0.651 |
| Barnesiella | 0.791 |  | -0.45 | 0.39 | 0.252 | 0.524 |
| Bifidobacterium | 0.904 |  | -0.18 | 0.25 | 0.473 | 0.756 |
| Bilophila | 0.791 |  | -0.06 | 0.22 | 0.805 | 0.907 |
| Brachyspira | 0.924 |  | 0.09 | 0.57 | 0.869 | 0.925 |
| Butyricicoccus | 0.904 |  | -0.27 | 0.09 | 0.004 | 0.028 |
| Campylobacter | 0.857 |  | 0.11 | 0.47 | 0.814 | 0.907 |
| Catenibacterium | 0.505 |  | -0.43 | 0.27 | 0.119 | 0.310 |
| Clostridium_IV | 0.904 |  | 0.04 | 0.17 | 0.819 | 0.907 |
| Clostridium_XI | 0.340 |  | -0.33 | 0.24 | 0.168 | 0.406 |
| Clostridium_XVIII | 0.783 |  | 0.13 | 0.48 | 0.792 | 0.907 |
| Collinsella | 0.267 |  | 0.07 | 0.12 | 0.535 | 0.783 |
| Corynebacterium | 0.924 |  | 0.72 | 0.30 | 0.018 | 0.072 |
| Desulfomicrobium | 0.924 |  | 0.00 | 0.19 | 0.990 | 0.990 |
| Desulfovibrio | 0.924 |  | -0.18 | 0.16 | 0.269 | 0.532 |
| Dialister | 0.445 |  | 0.13 | 0.10 | 0.196 | 0.453 |
| Dietzia | 0.924 |  | 1.10 | 0.40 | 0.007 | 0.030 |
| Enterorhabdus | 0.791 |  | -0.19 | 0.15 | 0.207 | 0.463 |
| Escherichia/Shigella | 0.924 |  | 0.34 | 0.43 | 0.429 | 0.726 |
| Eubacterium | 0.791 |  | 1.21 | 0.34 | 0.000 | 0.008 |
| Faecalibacterium | 0.857 |  | -0.28 | 0.08 | 0.001 | 0.011 |
| Finegoldia | 0.791 |  | 0.79 | 0.22 | 0.000 | 0.008 |
| Flavonifractor | 0.791 |  | 0.16 | 0.39 | 0.686 | 0.904 |
| Fusobacterium | 0.873 |  | 0.67 | 0.25 | 0.006 | 0.030 |
| Gardnerella | 0.873 |  | 0.86 | 0.63 | 0.171 | 0.406 |
| Gemmiger | 0.791 |  | -0.07 | 0.12 | 0.556 | 0.783 |
| Granulicatella | 0.924 |  | 0.42 | 0.40 | 0.301 | 0.581 |
| Haemophilus | 0.873 |  | 1.35 | 0.47 | 0.004 | 0.028 |
| Hallella | 0.857 |  | -0.20 | 0.35 | 0.563 | 0.783 |
| Helicobacter | 0.527 |  | 0.39 | 0.53 | 0.464 | 0.756 |
| Holdemania | 0.267 |  | 0.37 | 0.32 | 0.240 | 0.510 |
| Howardella | 0.305 |  | 0.03 | 0.13 | 0.841 | 0.907 |
| Hydrogenoanaerobacterium | 0.791 |  | -0.11 | 0.18 | 0.566 | 0.783 |
| Lactobacillus | 0.791 |  | -0.28 | 0.40 | 0.483 | 0.756 |
| Mannheimia | 0.001 | HIV- | -1.11 | 0.64 | 0.083 | 0.229 |
|  |  | HIV+ | 3.42 | 0.87 | 0.000 | 0.005 |
| Megasphaera | 0.873 |  | 0.00 | 0.14 | 0.984 | 0.990 |
| Methanosphaera | 0.402 |  | 0.31 | 0.35 | 0.380 | 0.686 |
| Mitsuokella | 0.857 |  | -0.03 | 0.15 | 0.814 | 0.907 |
| Mobiluncus | 0.924 |  | 0.23 | 0.33 | 0.479 | 0.756 |
| Mogibacterium | 0.873 |  | 0.50 | 0.22 | 0.021 | 0.079 |
| Murdochiella | 0.904 |  | 1.62 | 0.46 | 0.001 | 0.008 |
| Mycoplasma | 0.924 |  | -0.23 | 11.96 | 0.984 | 0.990 |
| Negativicoccus | 0.001 | HIV- | -0.72 | 0.34 | 0.033 | 0.119 |
|  |  | HIV+ | 1.65 | 0.43 | 0.000 | 0.005 |
| Oligosphaera | 0.106 |  | -0.33 | 0.35 | 0.349 | 0.651 |
| Olsenella | 0.549 |  | 0.21 | 0.14 | 0.149 | 0.374 |
| Oribacterium | 0.924 |  | 0.08 | 0.19 | 0.686 | 0.904 |
| Oscillibacter | 0.857 |  | 0.02 | 0.10 | 0.833 | 0.907 |
| Oxalobacter | 0.873 |  | -0.12 | 0.21 | 0.559 | 0.783 |
| Parabacteroides | 0.783 |  | 0.07 | 0.29 | 0.814 | 0.907 |
| Paraprevotella | 0.267 |  | -0.07 | 0.22 | 0.770 | 0.907 |
| Parasutterella | 0.924 |  | -0.28 | 0.43 | 0.512 | 0.778 |
| Parvimonas | 0.904 |  | 0.79 | 0.26 | 0.003 | 0.024 |
| Peptoniphilus | 0.913 |  | 0.66 | 0.20 | 0.001 | 0.012 |
| Peptostreptococcus | 0.924 |  | 0.59 | 0.21 | 0.006 | 0.030 |
| Porphyromonas | 0.791 |  | 0.64 | 0.26 | 0.015 | 0.064 |
| Prevotella | 0.857 |  | -0.03 | 0.08 | 0.733 | 0.907 |
| Pseudoflavonifractor | 0.791 |  | -0.30 | 0.35 | 0.397 | 0.701 |
| Ruminococcus | 0.857 |  | -0.36 | 0.18 | 0.051 | 0.164 |
| Slackia | 0.267 |  | -0.09 | 0.14 | 0.516 | 0.778 |
| Sneathia | 0.791 |  | -1.07 | 0.62 | 0.083 | 0.229 |
| Streptobacillus | 0.095 | HIV- | -2.08 | 1.07 | 0.051 | 0.164 |
|  |  | HIV+ | 1.06 | 0.35 | 0.002 | 0.024 |
| Streptococcus | 0.873 |  | 0.88 | 0.31 | 0.005 | 0.030 |
| Subdoligranulum | 0.924 |  | -0.07 | 0.21 | 0.723 | 0.907 |
| Succinivibrio | 0.627 |  | 0.10 | 0.41 | 0.807 | 0.907 |
| Sutterella | 0.791 |  | 0.07 | 0.23 | 0.756 | 0.907 |
| Turicibacter | 0.873 |  | -0.09 | 0.29 | 0.744 | 0.907 |
| Varibaculum | 0.857 |  | 0.27 | 0.34 | 0.422 | 0.726 |
| Veillonella | 0.791 |  | 1.19 | 0.50 | 0.018 | 0.072 |
| Victivallis | 0.873 |  | 0.16 | 0.35 | 0.652 | 0.887 |

Table S3. sparCC correlations between genera among n = 52 study participants testing positive for MA on urine drug screen. Correlations of absolute magnitude < 0.4 or FDR-adjusted *p* < .1 are not included.

| V1 | V2 | Corr | SE | p | q |
| --- | --- | --- | --- | --- | --- |
| Alloprevotella | Subdoligranulum | -0.562 | 0.05 | <.001 | <.001 |
| Alistipes | Ruminococcus | 0.461 | 0.105 | <.001 | <.001 |
| Anaerococcus | Porphyromonas | -0.413 | 0.077 | <.001 | <.001 |
| Alloprevotella | Peptostreptococcus | -0.434 | 0.084 | <.001 | <.001 |
| Anaerococcus | Peptostreptococcus | 0.501 | 0.09 | <.001 | <.001 |
| Alloprevotella | Peptoniphilus | 0.488 | 0.136 | <.001 | 0.004 |
| Butyricicoccus | Peptoniphilus | 0.412 | 0.132 | 0.002 | 0.017 |
| Butyricicoccus | Parvimonas | 0.445 | 0.113 | <.001 | 0.002 |
| Catenibacterium | Paraprevotella | -0.422 | 0.111 | <.001 | 0.002 |
| Acidaminococcus | Parabacteroides | 0.555 | 0.097 | <.001 | <.001 |
| Alloprevotella | Parabacteroides | 0.466 | 0.151 | 0.002 | 0.019 |
| Butyricicoccus | Parabacteroides | 0.474 | 0.129 | <.001 | 0.004 |
| Clostridium_IV | Parabacteroides | 0.557 | 0.066 | <.001 | <.001 |
| Anaerococcus | Oxalobacter | 0.482 | 0.114 | <.001 | 0.001 |
| Anaerococcus | Olsenella | 0.41 | 0.123 | 0.001 | 0.009 |
| Alloprevotella | Oligosphaera | -0.404 | 0.089 | <.001 | <.001 |
| Anaerococcus | Oligosphaera | 0.562 | 0.072 | <.001 | <.001 |
| Campylobacter | Oligosphaera | 0.512 | 0.103 | <.001 | <.001 |
| Dialister | Oligosphaera | 0.407 | 0.082 | <.001 | <.001 |
| Alloprevotella | Murdochiella | -0.446 | 0.083 | <.001 | <.001 |
| Anaerococcus | Murdochiella | 0.449 | 0.08 | <.001 | <.001 |
| Campylobacter | Murdochiella | 0.555 | 0.084 | <.001 | <.001 |
| Dietzia | Murdochiella | 0.605 | 0.072 | <.001 | <.001 |
| Acidaminococcus | Mogibacterium | 0.475 | 0.142 | 0.001 | 0.009 |
| Alloprevotella | Mogibacterium | 0.535 | 0.137 | <.001 | 0.002 |
| Butyricicoccus | Mogibacterium | 0.653 | 0.098 | <.001 | <.001 |
| Catenibacterium | Mogibacterium | 0.547 | 0.134 | <.001 | 0.001 |
| Clostridium_IV | Mogibacterium | 0.537 | 0.085 | <.001 | <.001 |
| Collinsella | Mogibacterium | 0.641 | 0.125 | <.001 | <.001 |
| Corynebacterium | Mogibacterium | -0.454 | 0.089 | <.001 | <.001 |
| Alloprevotella | Mobiluncus | -0.479 | 0.057 | <.001 | <.001 |
| Anaerococcus | Mobiluncus | 0.84 | 0.038 | <.001 | <.001 |
| Butyricicoccus | Mobiluncus | -0.426 | 0.092 | <.001 | <.001 |
| Corynebacterium | Mobiluncus | 0.591 | 0.1 | <.001 | <.001 |
| Dietzia | Mobiluncus | 0.511 | 0.075 | <.001 | <.001 |
| Faecalibacterium | Mobiluncus | -0.451 | 0.062 | <.001 | <.001 |
| Acetanaerobacterium | Mitsuokella | 0.412 | 0.094 | <.001 | <.001 |
| Alistipes | Mitsuokella | 0.426 | 0.096 | <.001 | <.001 |
| Finegoldia | Methanosphaera | 0.415 | 0.116 | <.001 | 0.005 |
| Anaerococcus | Mannheimia | -0.443 | 0.077 | <.001 | <.001 |
| Butyricicoccus | Mannheimia | 0.709 | 0.066 | <.001 | <.001 |
| Clostridium_IV | Mannheimia | 0.467 | 0.106 | <.001 | <.001 |
| Collinsella | Mannheimia | 0.458 | 0.133 | 0.001 | 0.007 |
| Faecalibacterium | Mannheimia | 0.73 | 0.064 | <.001 | <.001 |
| Finegoldia | Mannheimia | -0.48 | 0.09 | <.001 | <.001 |
| Fusobacterium | Mannheimia | -0.457 | 0.088 | <.001 | <.001 |
| Campylobacter | Howardella | 0.41 | 0.09 | <.001 | <.001 |
| Eubacterium | Howardella | 0.569 | 0.083 | <.001 | <.001 |
| Bulleidia | Haemophilus | 0.401 | 0.102 | <.001 | 0.001 |
| Campylobacter | Gemmiger | -0.416 | 0.094 | <.001 | <.001 |
| Escherichia.Shigella | Gemmiger | 0.434 | 0.119 | <.001 | 0.004 |
| Bifidobacterium | Gardnerella | 0.498 | 0.101 | <.001 | <.001 |
| Megasphaera | Flavonifractor | 0.584 | 0.101 | <.001 | <.001 |
| Alloprevotella | Finegoldia | -0.479 | 0.088 | <.001 | <.001 |
| Campylobacter | Finegoldia | 0.434 | 0.096 | <.001 | <.001 |
| Dietzia | Finegoldia | 0.475 | 0.101 | <.001 | <.001 |
| Eubacterium | Finegoldia | 0.533 | 0.104 | <.001 | <.001 |
| Hallella | Finegoldia | 0.409 | 0.128 | 0.001 | 0.014 |
| Campylobacter | Faecalibacterium | 0.516 | 0.105 | <.001 | <.001 |
| Dialister | Faecalibacterium | 0.418 | 0.126 | 0.001 | 0.01 |
| Eubacterium | Faecalibacterium | 0.421 | 0.092 | <.001 | <.001 |
| Alloprevotella | Eubacterium | -0.454 | 0.096 | <.001 | <.001 |
| Anaerococcus | Eubacterium | 0.41 | 0.081 | <.001 | <.001 |
| Campylobacter | Eubacterium | 0.522 | 0.084 | <.001 | <.001 |
| Dietzia | Eubacterium | 0.559 | 0.077 | <.001 | <.001 |
| Escherichia.Shigella | Eubacterium | -0.435 | 0.081 | <.001 | <.001 |
| Eubacterium | Eubacterium | 0.746 | 0.066 | <.001 | <.001 |
| Hallella | Eubacterium | 0.595 | 0.079 | <.001 | <.001 |
| Mobiluncus | Eubacterium | 0.475 | 0.121 | <.001 | 0.002 |
| Mogibacterium | Eubacterium | 0.446 | 0.085 | <.001 | <.001 |
| Gardnerella | Escherichia.Shigella | 0.515 | 0.13 | <.001 | 0.001 |
| Gemmiger | Escherichia.Shigella | -0.403 | 0.096 | <.001 | 0.001 |
| Dietzia | Enterorhabdus | 0.403 | 0.105 | <.001 | 0.002 |
| Finegoldia | Enterorhabdus | 0.409 | 0.082 | <.001 | <.001 |
| Acidaminococcus | Desulfomicrobium | 0.417 | 0.099 | <.001 | 0.001 |
| Alistipes | Desulfomicrobium | 0.432 | 0.091 | <.001 | <.001 |
| Butyricicoccus | Desulfomicrobium | 0.503 | 0.11 | <.001 | <.001 |
| Clostridium_IV | Desulfomicrobium | 0.51 | 0.09 | <.001 | <.001 |
| Collinsella | Desulfomicrobium | 0.462 | 0.11 | <.001 | 0.001 |
| Corynebacterium | Desulfomicrobium | -0.429 | 0.078 | <.001 | <.001 |
| Faecalibacterium | Desulfomicrobium | 0.712 | 0.057 | <.001 | <.001 |
| Gemmiger | Desulfomicrobium | 0.518 | 0.093 | <.001 | <.001 |
| Alistipes | Collinsella | 0.536 | 0.095 | <.001 | <.001 |
| Bacteroides | Collinsella | 0.435 | 0.109 | <.001 | 0.001 |
| Bilophila | Collinsella | 0.48 | 0.105 | <.001 | <.001 |
| Butyricicoccus | Collinsella | 0.421 | 0.114 | <.001 | 0.003 |
| Faecalibacterium | Collinsella | 0.408 | 0.109 | <.001 | 0.003 |
| Finegoldia | Collinsella | -0.435 | 0.094 | <.001 | <.001 |
| Flavonifractor | Collinsella | 0.533 | 0.085 | <.001 | <.001 |
| Gemmiger | Collinsella | 0.468 | 0.099 | <.001 | <.001 |
| Oscillibacter | Collinsella | 0.445 | 0.102 | <.001 | <.001 |
| Actinomyces | Clostridium_IV | 0.451 | 0.103 | <.001 | <.001 |
| Fusobacterium | Clostridium_IV | 0.411 | 0.124 | 0.001 | 0.01 |
| Granulicatella | Clostridium_IV | 0.548 | 0.116 | <.001 | <.001 |
| Actinomyces | Catenibacterium | 0.447 | 0.1 | <.001 | <.001 |
| Alloprevotella | Catenibacterium | -0.54 | 0.056 | <.001 | <.001 |
| Anaerococcus | Catenibacterium | 0.831 | 0.053 | <.001 | <.001 |
| Butyricicoccus | Catenibacterium | -0.486 | 0.077 | <.001 | <.001 |
| Campylobacter | Catenibacterium | 0.486 | 0.108 | <.001 | <.001 |
| Corynebacterium | Catenibacterium | 0.466 | 0.109 | <.001 | <.001 |
| Dietzia | Catenibacterium | 0.595 | 0.074 | <.001 | <.001 |
| Eubacterium | Catenibacterium | 0.522 | 0.077 | <.001 | <.001 |
| Faecalibacterium | Catenibacterium | -0.496 | 0.054 | <.001 | <.001 |
| Finegoldia | Catenibacterium | 0.848 | 0.033 | <.001 | <.001 |
| Flavonifractor | Catenibacterium | -0.447 | 0.096 | <.001 | <.001 |
| Gemmiger | Catenibacterium | -0.505 | 0.075 | <.001 | <.001 |
| Murdochiella | Catenibacterium | 0.491 | 0.08 | <.001 | <.001 |
| Parabacteroides | Catenibacterium | -0.468 | 0.101 | <.001 | <.001 |
| Parvimonas | Catenibacterium | 0.482 | 0.096 | <.001 | <.001 |
| Actinomyces | Campylobacter | 0.416 | 0.094 | <.001 | <.001 |
| Anaerococcus | Campylobacter | 0.538 | 0.095 | <.001 | <.001 |
| Finegoldia | Campylobacter | 0.575 | 0.078 | <.001 | <.001 |
| Fusobacterium | Campylobacter | 0.496 | 0.114 | <.001 | <.001 |
| Gemmiger | Campylobacter | -0.464 | 0.093 | <.001 | <.001 |
| Granulicatella | Campylobacter | 0.618 | 0.088 | <.001 | <.001 |
| Parabacteroides | Campylobacter | -0.426 | 0.101 | <.001 | 0.001 |
| Parvimonas | Campylobacter | 0.798 | 0.063 | <.001 | <.001 |
| Peptoniphilus | Campylobacter | 0.612 | 0.086 | <.001 | <.001 |
| Alloprevotella | Butyricicoccus | -0.507 | 0.074 | <.001 | <.001 |
| Anaerococcus | Butyricicoccus | 0.502 | 0.099 | <.001 | <.001 |
| Butyricicoccus | Butyricicoccus | -0.403 | 0.096 | <.001 | 0.001 |
| Campylobacter | Butyricicoccus | 0.461 | 0.116 | <.001 | 0.001 |
| Collinsella | Butyricicoccus | -0.442 | 0.092 | <.001 | <.001 |
| Dietzia | Butyricicoccus | 0.484 | 0.087 | <.001 | <.001 |
| Eubacterium | Butyricicoccus | 0.606 | 0.076 | <.001 | <.001 |
| Mobiluncus | Butyricicoccus | 0.459 | 0.089 | <.001 | <.001 |
| Murdochiella | Butyricicoccus | 0.552 | 0.084 | <.001 | <.001 |
| Peptoniphilus | Butyricicoccus | 0.569 | 0.081 | <.001 | <.001 |
| Clostridium_IV | Bilophila | 0.612 | 0.074 | <.001 | <.001 |
| Collinsella | Bilophila | 0.44 | 0.103 | <.001 | <.001 |
| Faecalibacterium | Bilophila | 0.414 | 0.143 | 0.004 | 0.029 |
| Oscillibacter | Bilophila | 0.437 | 0.112 | <.001 | 0.002 |
| Parvimonas | Bilophila | -0.412 | 0.108 | <.001 | 0.002 |
| Peptostreptococcus | Bilophila | -0.44 | 0.103 | <.001 | <.001 |
| Gardnerella | Barnesiella | 0.45 | 0.128 | <.001 | 0.006 |
| Mycoplasma | Barnesiella | 0.435 | 0.14 | 0.002 | 0.018 |
| Gardnerella | Bacteroides | 0.527 | 0.12 | <.001 | <.001 |
| Mycoplasma | Bacteroides | 0.416 | 0.14 | 0.003 | 0.025 |
| Parvimonas | Bacteroides | 0.439 | 0.089 | <.001 | <.001 |
| Peptostreptococcus | Bacteroides | 0.438 | 0.074 | <.001 | <.001 |
| Escherichia.Shigella | Arcanobacterium | 0.5 | 0.112 | <.001 | <.001 |
| Mogibacterium | Arcanobacterium | -0.448 | 0.095 | <.001 | <.001 |
| Acetanaerobacterium | Alloprevotella | -0.414 | 0.081 | <.001 | <.001 |
| Clostridium_XI | Alistipes | 0.449 | 0.097 | <.001 | <.001 |
| Anaerococcus | Actinomyces | 0.459 | 0.074 | <.001 | <.001 |
| Dietzia | Actinomyces | 0.496 | 0.092 | <.001 | <.001 |
| Eubacterium | Actinomyces | 0.595 | 0.089 | <.001 | <.001 |
| Hallella | Actinomyces | 0.413 | 0.119 | 0.001 | 0.006 |
| Murdochiella | Actinomyces | 0.597 | 0.099 | <.001 | <.001 |
| Peptoniphilus | Actinomyces | 0.507 | 0.075 | <.001 | <.001 |
| Haemophilus | Acidaminococcus | 0.509 | 0.115 | <.001 | <.001 |
| Mogibacterium | Acidaminococcus | -0.408 | 0.098 | <.001 | 0.001 |
| Streptococcus | Acidaminococcus | 0.42 | 0.087 | <.001 | <.001 |
| Methanosphaera | Acetanaerobacterium | 0.446 | 0.119 | <.001 | 0.003 |
| Oligosphaera | Acetanaerobacterium | 0.477 | 0.099 | <.001 | <.001 |

Figure S1. **Rectal microbial composition of study participants, stratified by methamphetamine (MA) use and HIV status.**


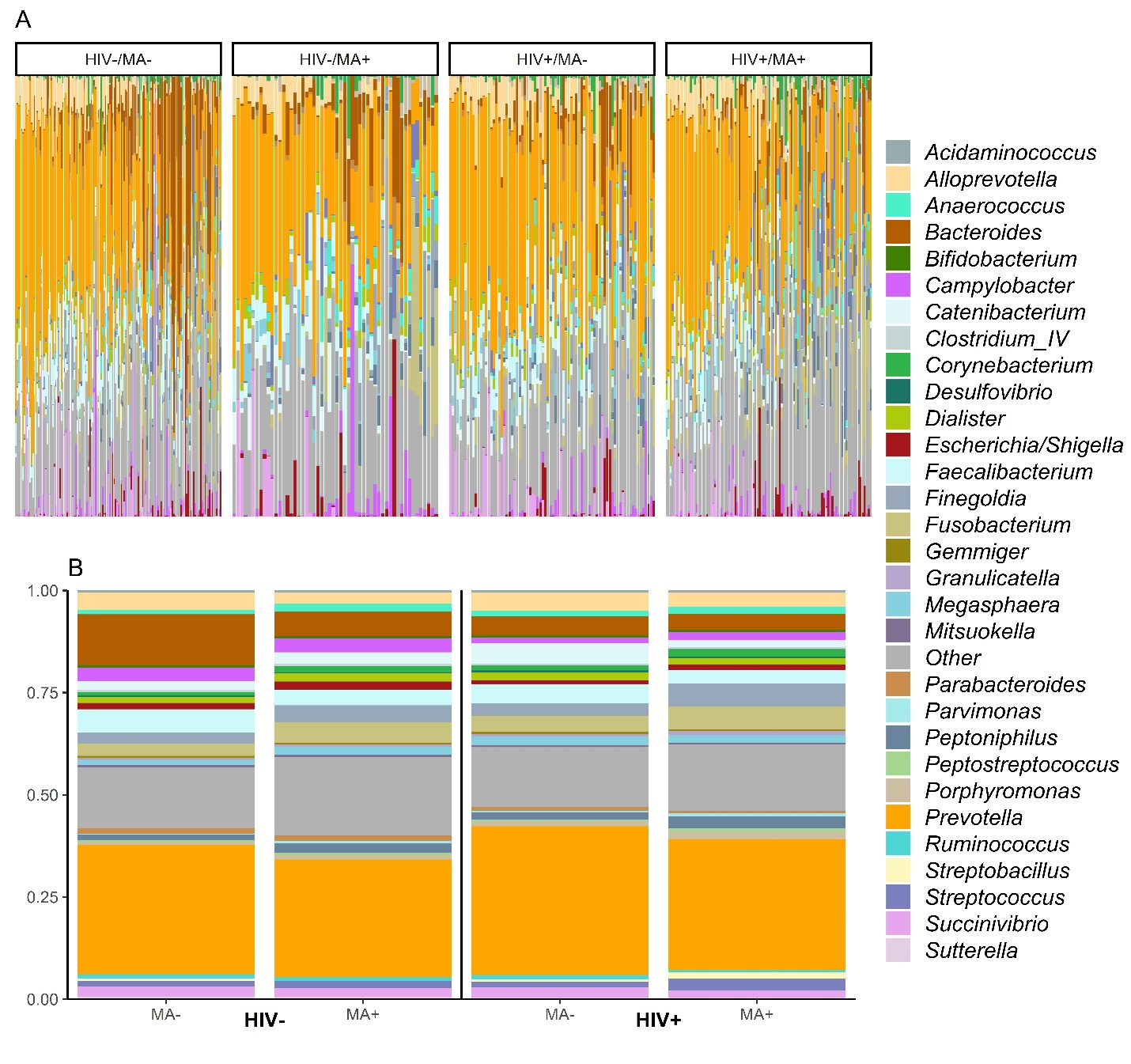


Figure S2. **Associations between methamphetamine (MA) use and overall microbial composition and diversity, stratified by HIV status.**


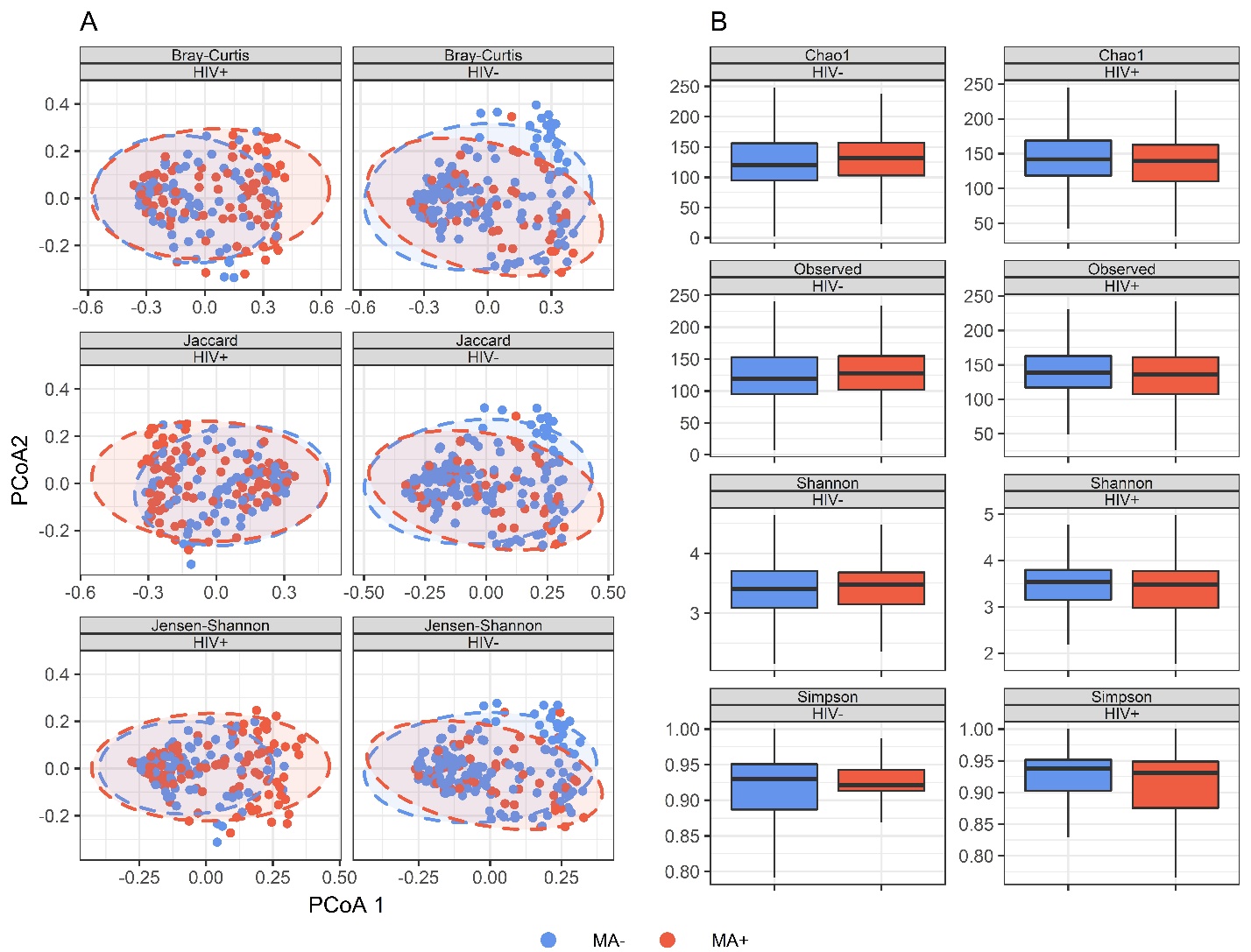


Figure S3. **Dose-response analyses of bacterial counts on increasing frequency of methamphetamine (MA) use.** Plots show model-fitted log bacterial counts at each level of MA use with linear dose-response curves drawn to fit the points. Model-fitted values were calculated using coefficients from the count portion of zero-inflated negative binomial (ZINB) models at the sample-average value of covariates. Significance of the curves was assessed using ZINB models with orthogonal polynomial coding of MA use. Quadratic curves were also calculated, but were not significant, and are not shown.


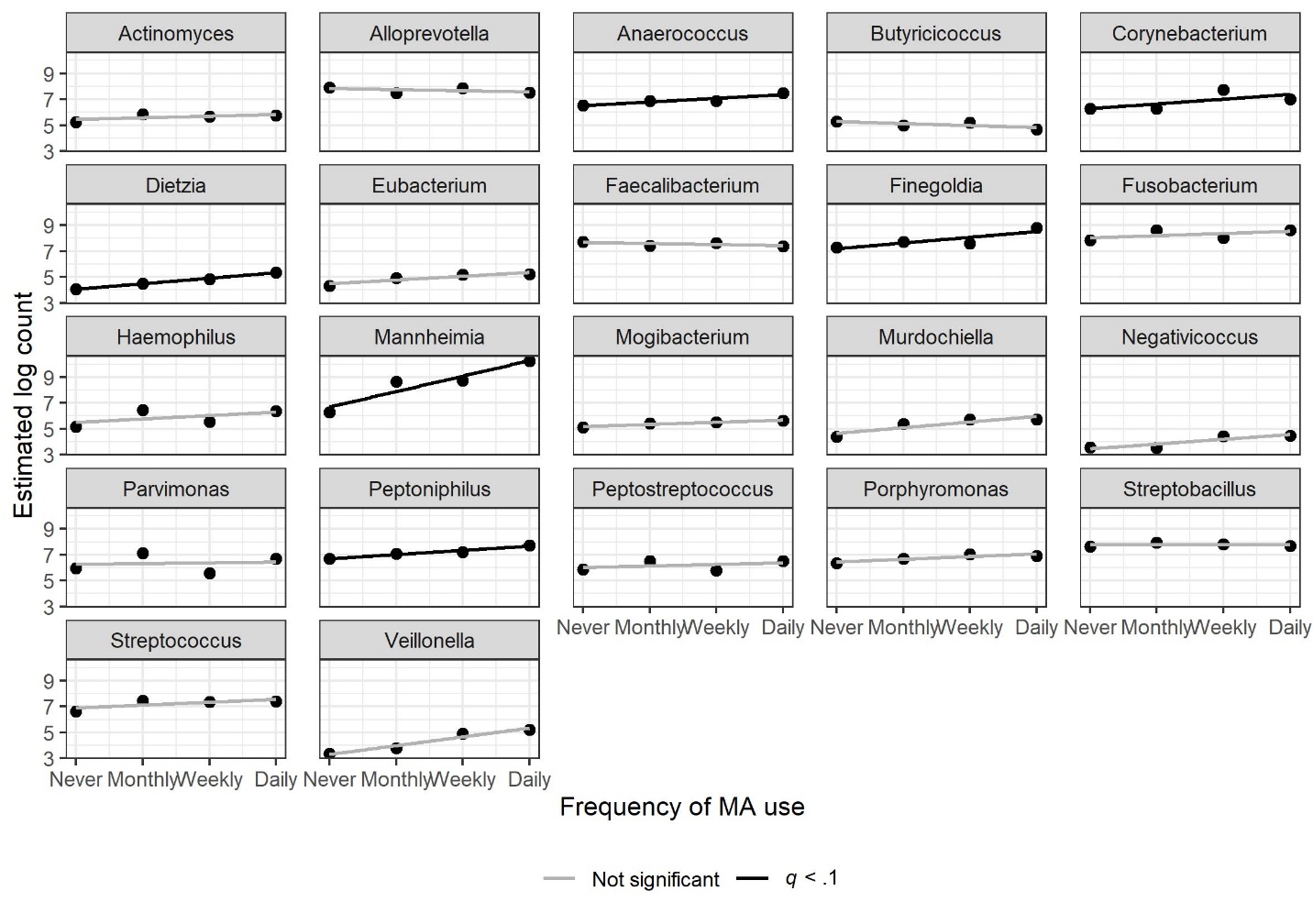


Figure S4. **Replication of Figure 1, with participants’ MA status determined by urine drug screen.**


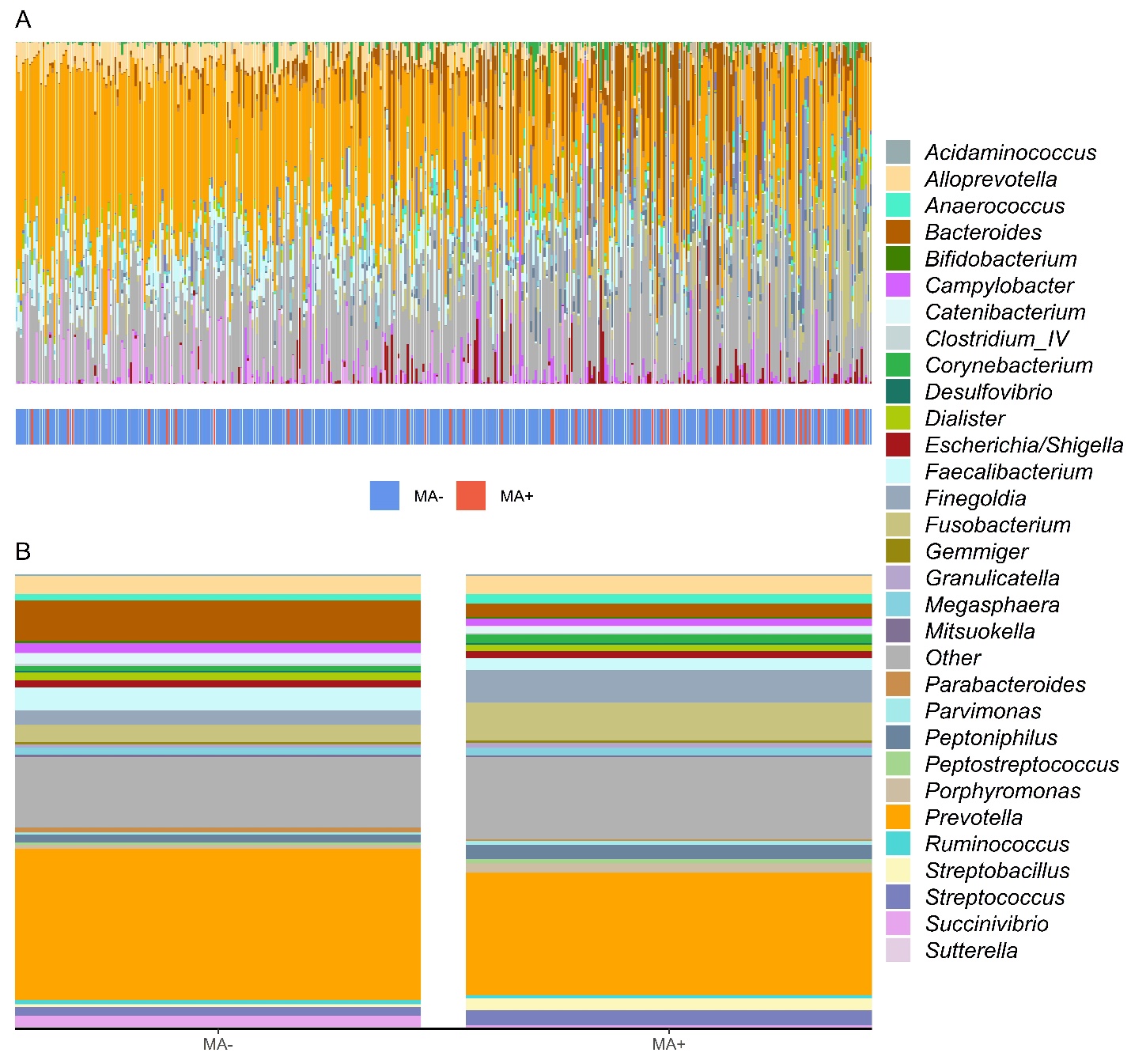


Figure S5. **Replication of Figure 2, with participants’ MA status determined by urine drug screen.**

**
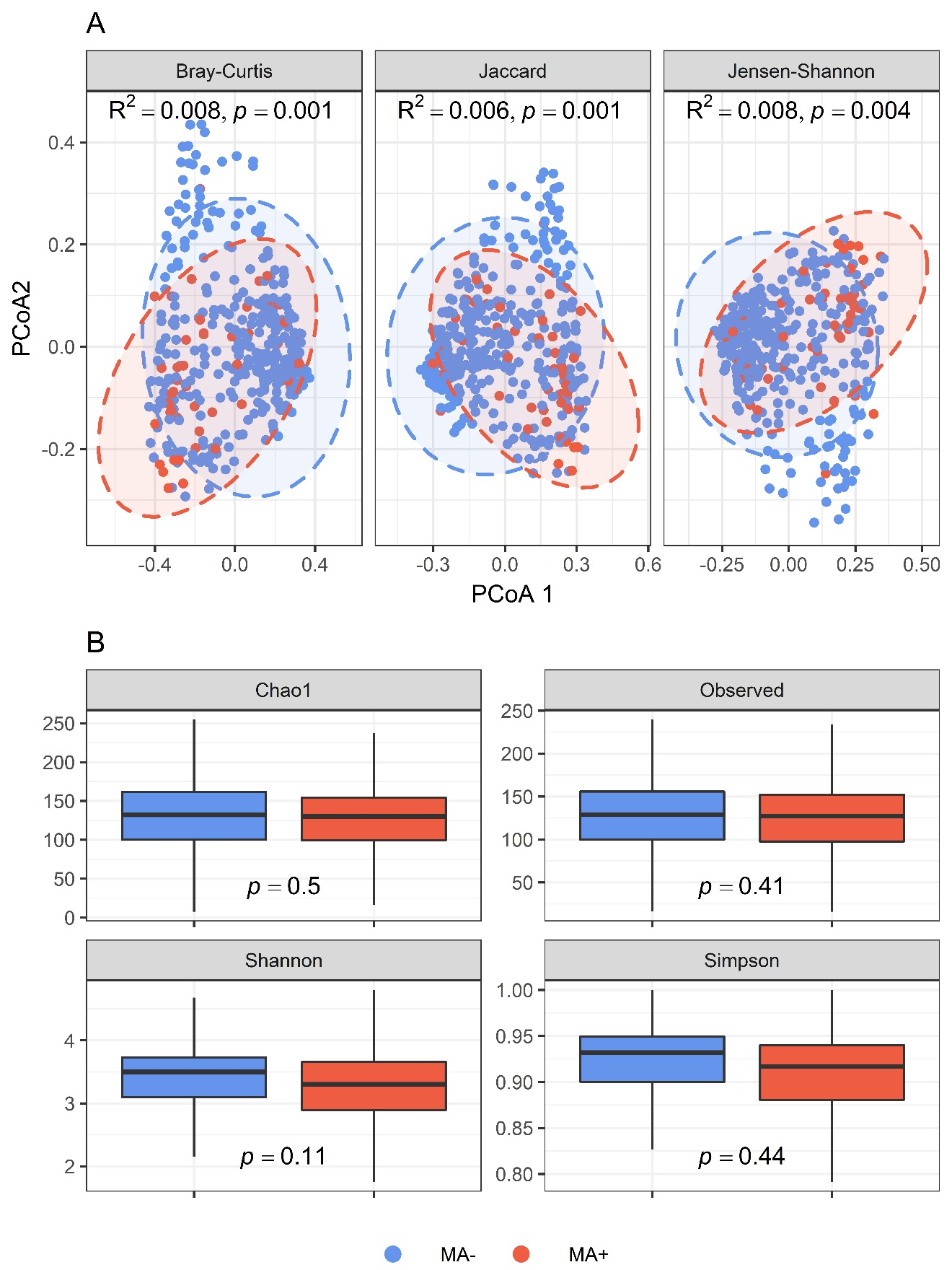
**

Figure S6. **Replication of Figure 3, with participants’ MA status determined by urine drug screen.**

**
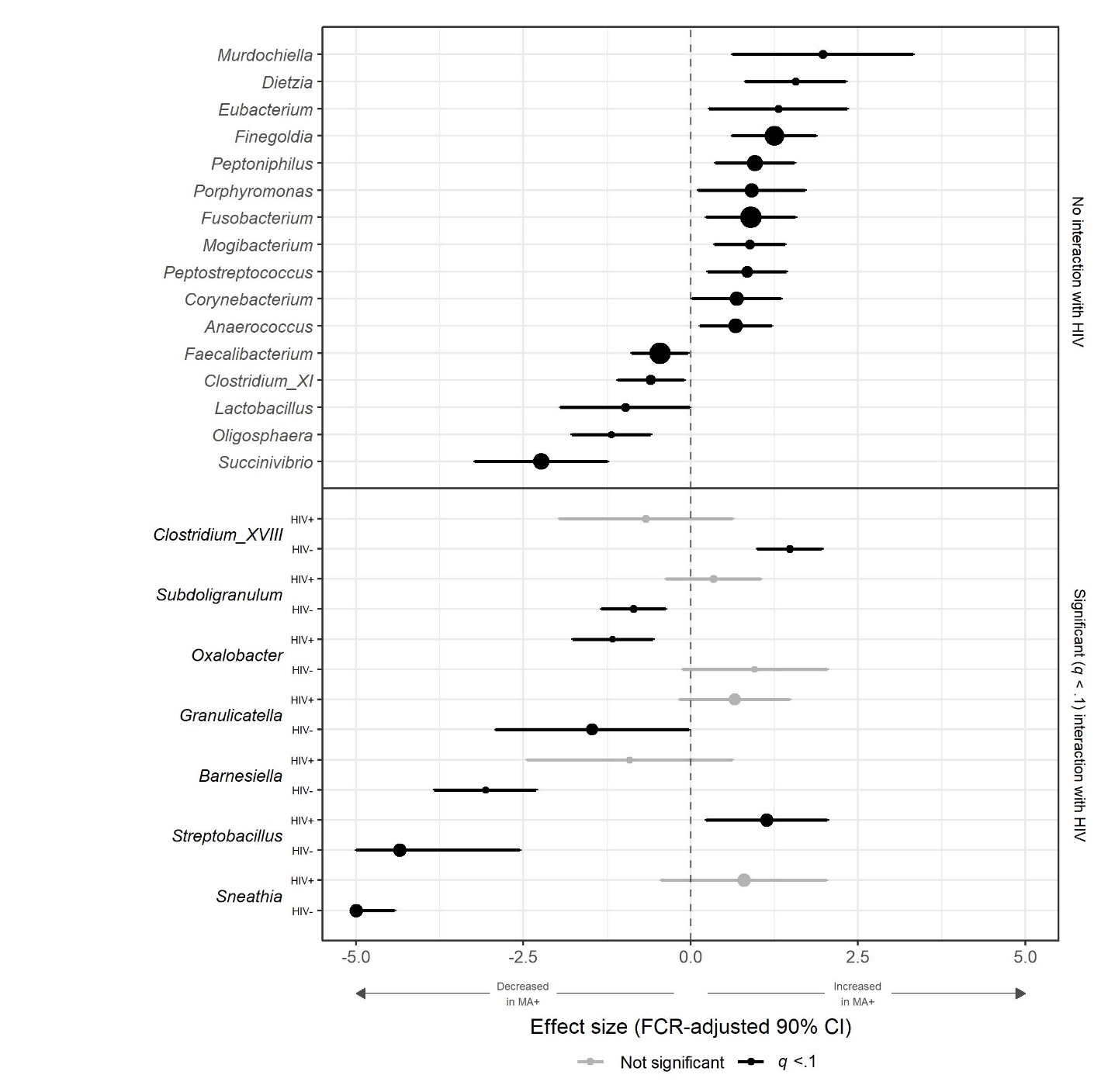
**

Figure S7. **sparCC correlations between genera among n = 52 study participants testing positive for MA on urine drug screen**. Correlations of absolute magnitude < 0.4 or FDR-adjusted *p* < .1 are not shown.


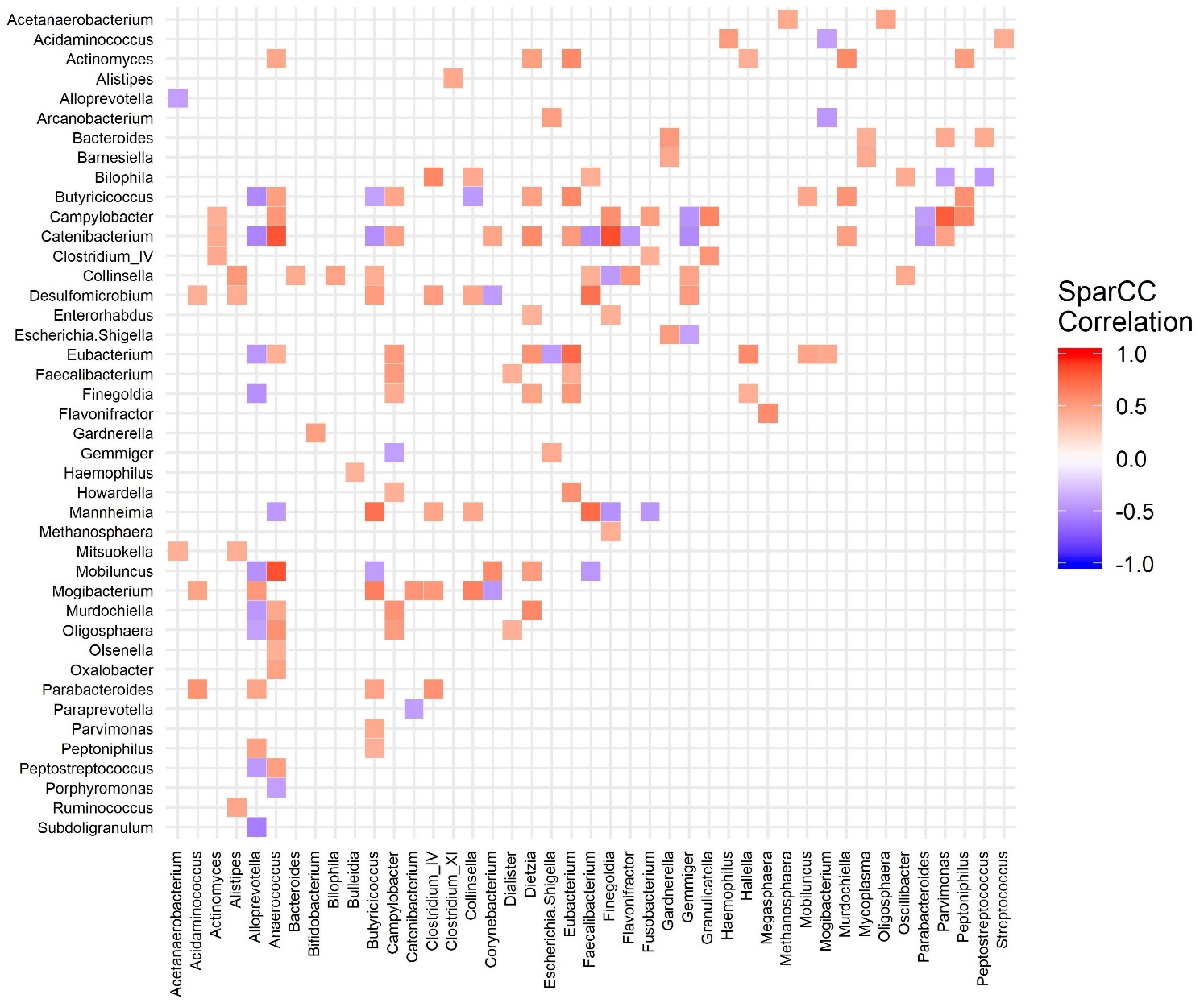


References

1 Robins, J. M., Hernan, M. A. & Brumback, B. Marginal structural models and causal inference in epidemiology. *Epidemiology (Cambridge, Mass.)* **11**, 550-560 (2000).

2 Babyak, M. A. What you see may not be what you get: a brief, nontechnical introduction to overfitting in regression-type models. *Psychosomatic medicine* **66**, 411-421 (2004).

3 De Stavola, B. L. & Cox, D. R. On the consequences of overstratification. *Biometrika* **95**, 992-996, doi:10.1093/biomet/asn039 (2008).

4 VanderWeele, T. J. On the distinction between interaction and effect modification. *Epidemiology (Cambridge, Mass.)* **20**, 863-871, doi:10.1097/EDE.0b013e3181ba333c (2009).

5 McCaffrey, D. F. *et al.* A tutorial on propensity score estimation for multiple treatments using generalized boosted models. *Statistics in medicine* **32**, 3388-3414, doi:10.1002/sim.5753 (2013).

6 Normand, S. T. *et al.* Validating recommendations for coronary angiography following acute myocardial infarction in the elderly: a matched analysis using propensity scores. *J Clin Epidemiol* **54**, 387-398 (2001).

7 Austin, P. C. Balance diagnostics for comparing the distribution of baseline covariates between treatment groups in propensity-score matched samples. *Statistics in medicine* **28**, 3083-3107, doi:10.1002/sim.3697 (2009).

8 Panek, M. *et al.* Methodology challenges in studying human gut microbiota – effects of collection, storage, DNA extraction and next generation sequencing technologies. *Scientific reports* **8**, 5143, doi:10.1038/s41598-018-23296-4 (2018).
